# Qinghai-like H5N1 from Domestic Cats, Northern Iraq

**DOI:** 10.3201/eid1208.060264

**Published:** 2006-08

**Authors:** Samuel L. Yingst, Magdi D. Saad, Stephen A. Felt

**Affiliations:** * US Naval Medical Research Unit No. 3, Cairo, Egypt

**Keywords:** Avian Influenza, H5N1, Cat, Goose, Human, Zoonoses, Sentinel, letter

To the Editor: Natural infection of several cat species with highly pathogenic avian influenza (HPAI) H5N1 viruses in Thailand (*1**–**4*) and experimental infection of domestic cats with similar viruses have been reported (*5**,**6*). Thus, literature describing HPAI H5N1 infection of cats is limited to descriptions of infections with a subset of clade I viruses. HPAI H5N1 viruses, highly similar to viruses isolated from Qinghai Lake in western People's Republic of China in spring 2005, are now rapidly disseminating throughout Eurasia and Africa. To our knowledge, this is the first report of a Qinghai-like virus detected in domestic cats. This finding is noteworthy because the host range of influenza viruses is determined by the antigenic characteristics of the hemagglutinin and neuraminidase molecules; clade II viruses are antigenically distinct from clade I viruses, and Qinghai-like viruses are genetically distinct from other clade II viruses.

Personal communications in January 2006 from field veterinarians noted deaths of domestic cats that were associated with suspected (eventually confirmed) H5N1 outbreaks in eastern Turkey (2 villages) and Kurdish northern Iraq (Sarcapcarn in Sulymaniyah Governorate and Grd Jotyar in Erbil Governorate). The clinical conditions of the birds did not suggest HPAI to villagers or consulting veterinarians. In both scenarios in Iraq, results of rapid antigen detection tests with the Anigen kit (Suwon, Republic of Korea), while positive for influenza A, were negative for H5, so the outbreaks were not thought to be caused by HPAI, but concern about the unusual deaths in cats remained. Because the regions are remote and veterinary services limited, such anecdotal reports have rarely been followed up.

After H5N1 influenza was diagnosed in a person in Sarcapcarn, Kurdish northern Iraq, the government of Iraq requested a World Health Organization investigation, which was supported in part by Naval Medical Research Unit No. 3 veterinarians. While investigating the situation in Erbil Governorate, the team was informed of suspicious deaths in cats associated with the death of all 51 chickens in a household in Grd Jotyar (≈10 km north of Erbil City) From February 3 to February 5, 2006, five cats reportedly died; 2 of these were available for examination on February 8. A sick goose from an adjacent household was killed and underwent necropsy with the cats. Gross pathologic changes in cats were similar to those previously reported, except that severe hemorrhagic pancreatitis was observed (*5**,**6*). Tissues from these animals and archived tissues from 1 of the 51 chickens were conveyed to Cairo for virologic examination.

An influenza A H5 virus was present in multiple organs in all species from the outbreak site in Grd Jotyar (Table). cDNA for sequencing was amplified directly from RNA extracts from pathologic materials without virus isolation. On the basis of sequence analysis of the full HA1 gene and 219 amino acids of the HA2 gene, the viruses from the goose and 1 cat from Grd Jotyar and from the person who died from Sarcapcarn (sequence derived from PCR amplification from first-passage egg material) are >99% identical at the nucleotide and amino acid levels (GenBank nos. DQ435200–02). Thus, no indication of virus adaptation to cats was found. The viruses from Iraq are most closely related to currently circulating Qinghai-like viruses, but when compared with A/bar-headed goose/Qinghai/65/2005 (H5N1) (GenBank no. DQ095622), they share only 97.4% identity at the nucleic acid level with 3 amino acid substitutions of unknown significance. On the other hand, the virus from the cat is only 93.4% identical to A/tiger/Thailand/CU-T4/2004(H5N1) (GenBank no. AY972539). These results are not surprising, given that these strains are representative of different clades (*8**,**9*). Sequencing of 1,349 bp of the N gene from cat 1 and the goose (to be submitted to GenBank) show identity at the amino acid level, and that the N genes of viruses infecting the cat and goose are >99% identical to that of A/bar-headed goose/Qinghai/65/2005(H5N1). These findings support the notion that cats may be broadly susceptible to circulating H5N1 viruses and thus may play a role in reassortment, antigenic drift, and transmission.

**Table:** Detection of influenza A H5 by real-time PCR*

Tissue	Chicken	Goose	Cat 1	Cat 2
Abdominal fluid	ND	+	ND	ND
Blood	ND	–	–	ND
Heart	+	ND	ND	ND
Trachea	ND	–	+	+
Lung	+	+	+	+
Kidney	ND	ND	–	ND
Spleen	ND	–	–	–
Pancreas	ND	ND	+	+
Lymph node	ND	ND	–	ND
Liver	+	ND	–	+
Proventriculus	+	ND	N/A	N/A
Small intestine	+	+	–	ND
Large intestine	ND	–	+	+
Cecum	ND	+	ND	ND
Feces	ND	ND	+	ND

*ND, not done. Samples were tested by real time PCR for influenza A (matrix protein) and if positive, for H5 (*7*). All samples denoted as positive were positive for both influenza A and H5. Chicken samples were obtained previously by local veterinarians based on their sampling protocols. Goose and cat samples were obtained by S. Felt; only grossly abnormal tissues were sampled.

The route of infection in these cats cannot be determined definitively. How cats behave when eating birds makes both oral and respiratory infection possible. However, the source of infection seems clear in that an identical H5N1 virus was detected in samples from a goose from the same dwelling, and an H5 virus was detected from archived samples from 1 of 51 chickens that died over the course of a few days. The potential for horizontal spread cannot be ruled out since we detected viral RNA in gut, stool, and trachea; clinical signs developed in all cats, and all died from the acute illness 2–4 days after the chicken deaths began; therefore, simultaneous exposure seems likely. Death in cats, spatially and temporally associated with unusual deaths in poultry, especially when the cats show positive results of a rapid antigen detection test for influenza A, should be considered to indicate a presumptive diagnosis of HPAI, and appropriate response should ensue.
